# Supplementary material for: Formation of Superhydrophobic Coatings Based on Dispersion Compositions of Hexyl Methacrylate Copolymers with Glycidyl Methacrylate and Silica Nanoparticles
Source: Polymers (Basel). 2024 Nov 1;16(21):3094. doi: 10.3390/polym16213094 (PMC11548060; doi:10.3390/polym16213094)
Supplement: Supplementary file 1 [file polymers-16-03094-s001.zip › polymers-3249207-supplementary.pdf]

Table S1 – Results of measurements of average initial wetting angles and average roll-off angles for composite coatings based on poly-(HMA-co-GMA).

No.	Concentration of copolymer in the organic phase, wt.%	Filler/polymer binder mass fraction ratio, W_f/W_p	Contact angle, deg	Roll-off angle, deg
1	5	0	91.65±1.5	-
2		0.4	152.95±2.5	12.29±1.5
3		0.8	156.64±2	9.96±1
4		1.2	157.39±3	6.46±1
5		1.6	155.46±2.5	6.1±1
6		2	152.94±2.5	6.99±1
7	10	0	91.77±1.5	-
8		0.4	150.09±2	17.1±1.5
9		0.8	155.36±3	10.26±1.2
10		1.2	154.11±2.5	9.97±1
11		1.6	156.91±2	13.79±1.5
12		2	158.94±3	18.12±2
13	15	0	89.77±2	
14		0.4	153.56±2.5	20.07±2.5
15		0.8	155.24±2	10.7±1
16		1.2	155.85±3	9.93±1
17		1.6	155.43±2.5	21.05±2
19	20	0	92.63±1	-
20		0.4	105.76±2.5	-
21		0.8	114.26±3	-
22		1.2	117.31±2.5	-
23		1.6	145.46±2	-

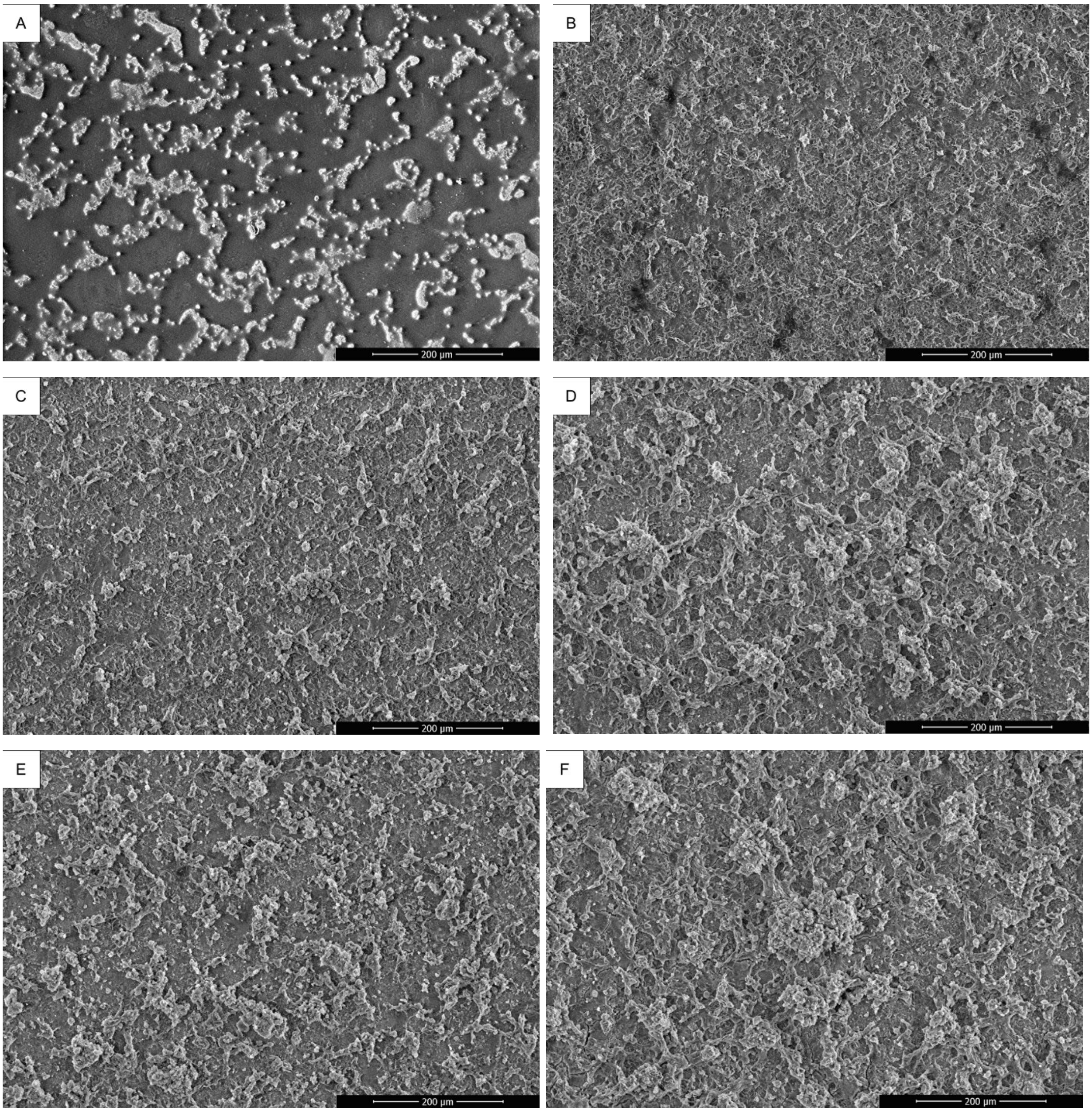

Figure S1 – SEM images of coatings based on a 5 wt.% poly(HMA-co-GMA) solution. showing variations in the filler/polymer binder ratios: A – 0; B – 0.4; C – 0.8; D – 1.2; E – 1.6; F – 2.0 (500× magnification)

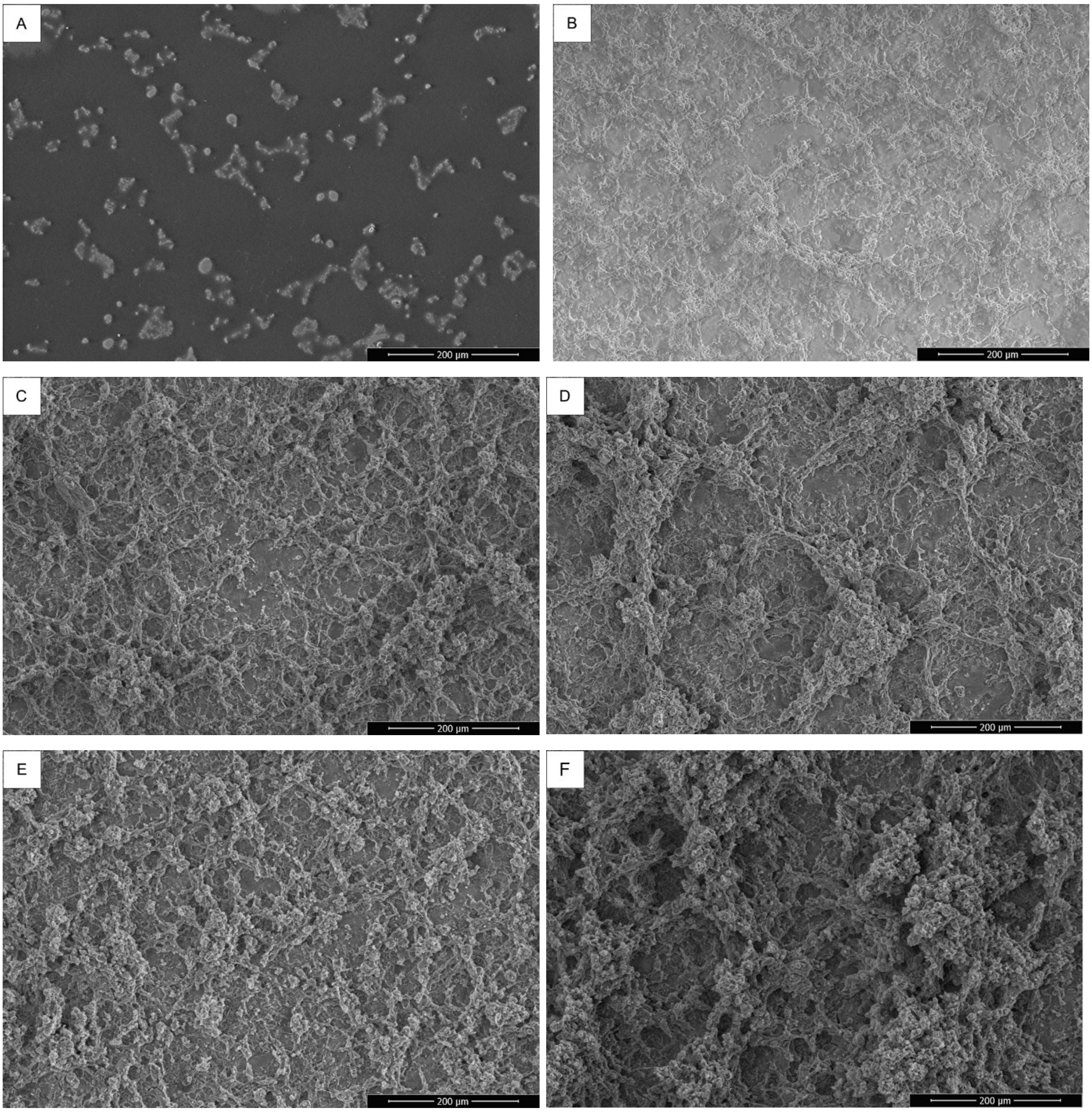

Figure S2 – SEM images of coatings based on a 10 wt.% poly(HMA-co-GMA) solution. showing variations in the filler/polymer binder ratios: A – 0; B – 0.4; C – 0.8; D – 1.2; E – 1.6; F – 2.0 (500× magnification)

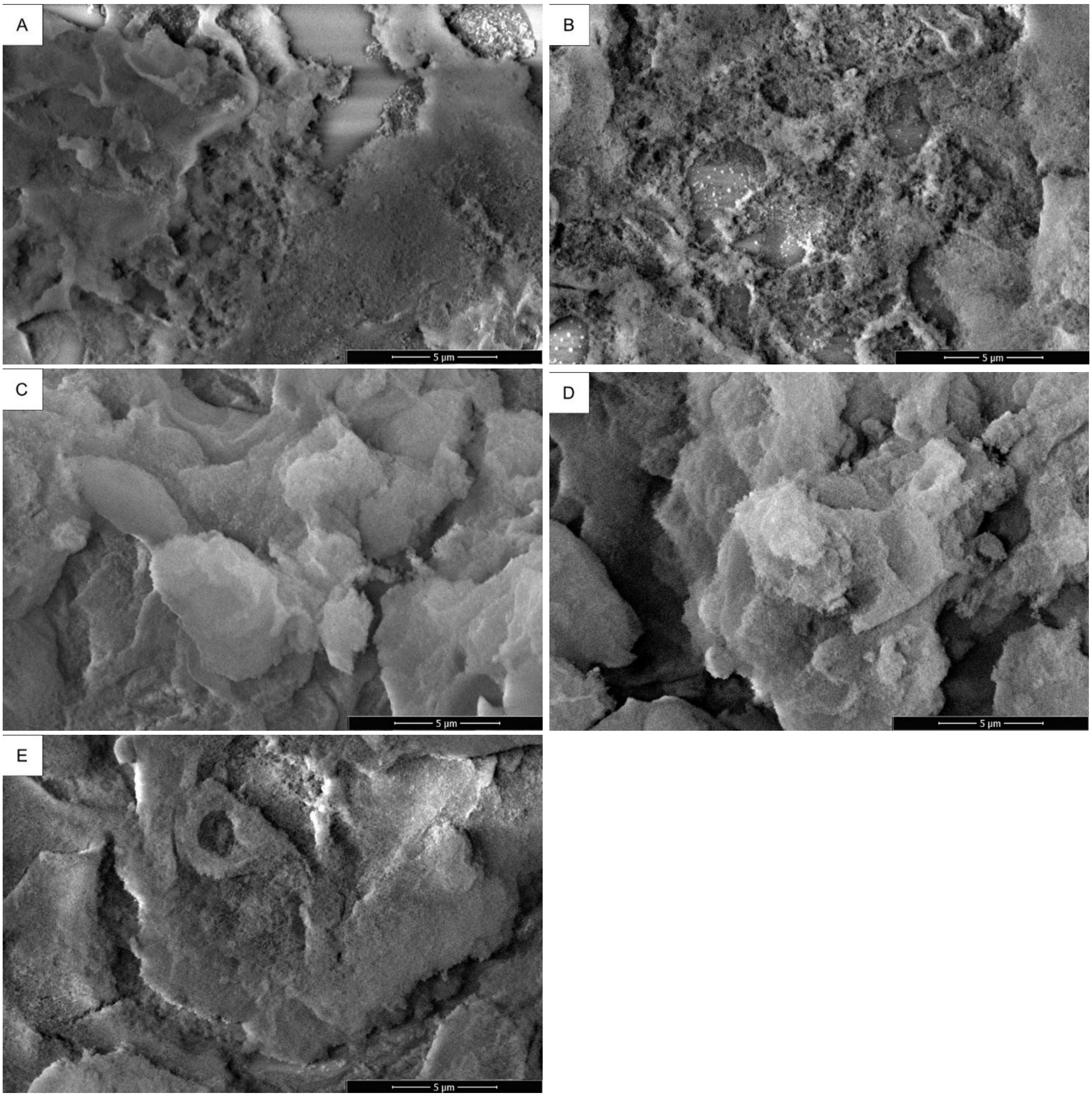

Figure S3 – SEM images of coatings based on a 5 wt.% poly(HMA-co-GMA) solution. showing variations in the filler/polymer binder ratios: A – 0.4; B – 0.8; C – 1.2; D – 1.6; E – 2.0 (16000× magnification)

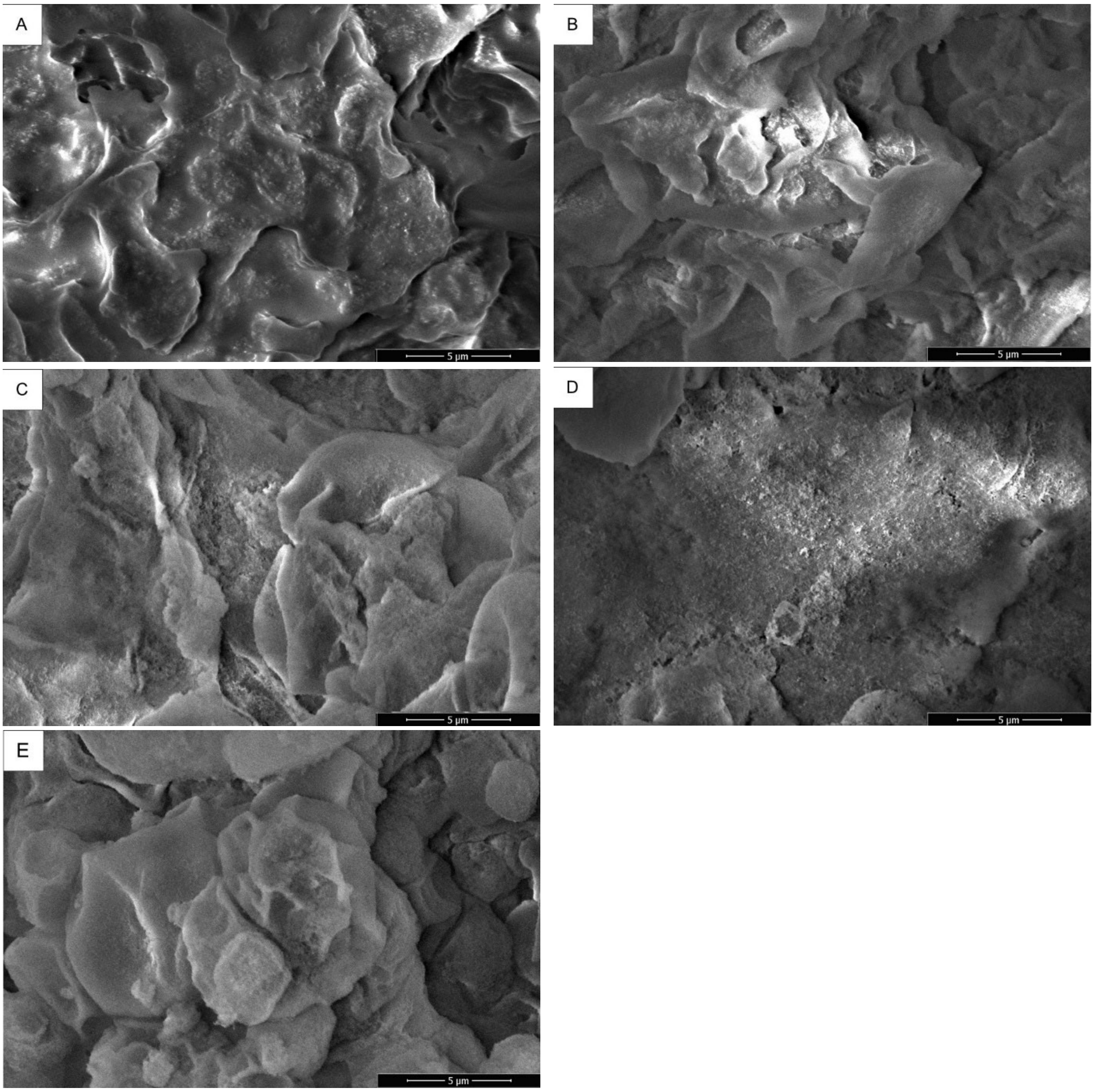

Figure S4 – SEM images of coatings based on a 10 wt.% poly(HMA-co-GMA) solution. showing variations in the filler/polymer binder ratios: A – 0.4; B – 0.8; C – 1.2; D – 1.6; E – 2.0 (16000× magnification)

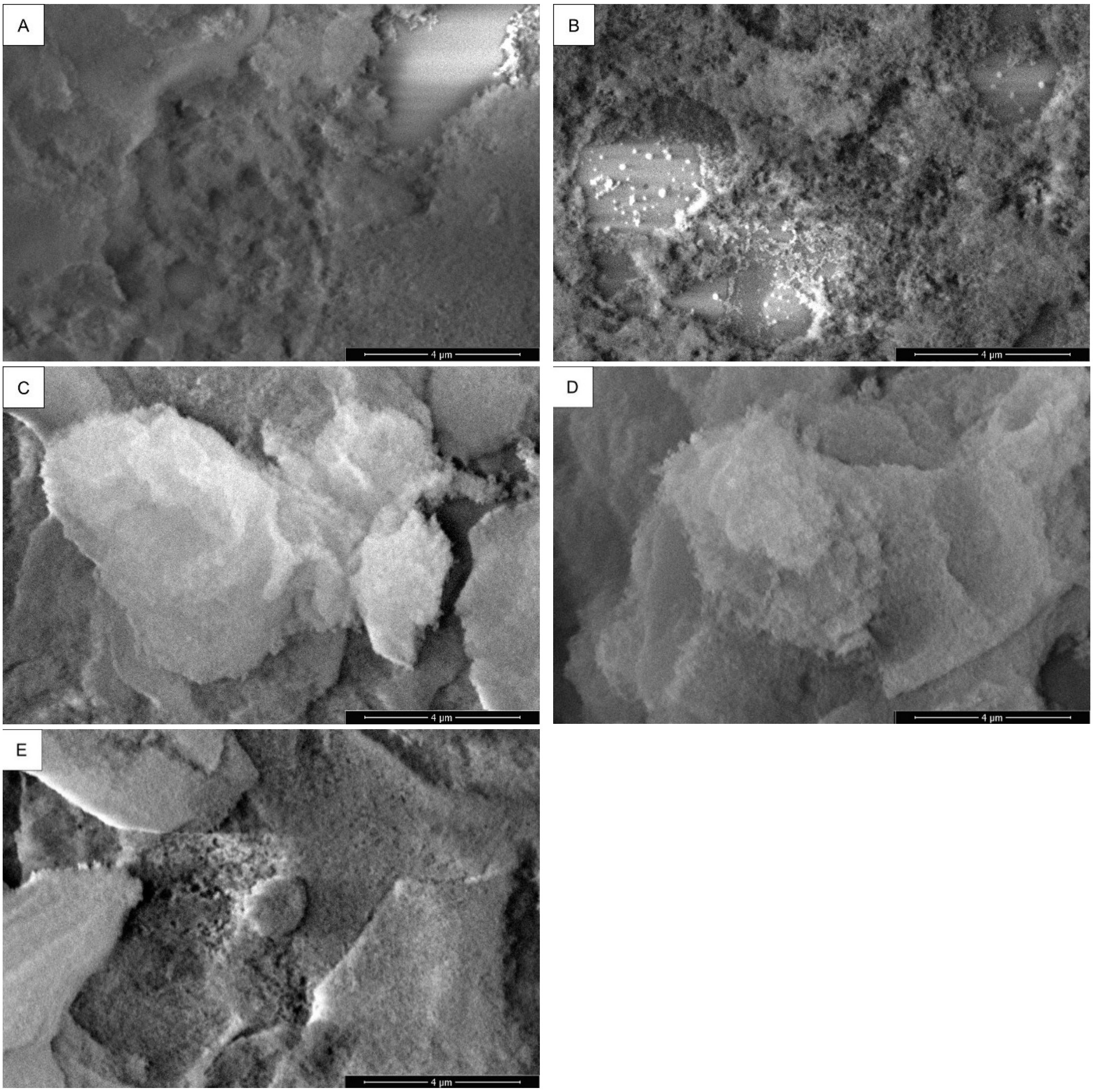

Figure S5 – SEM images of coatings based on a 5 wt.% poly(HMA-co-GMA) solution. showing variations in the filler/polymer binder ratios: A – 0.4; B – 0.8; C – 1.2; D – 1.6; E – 2.0 (30000× magnification)

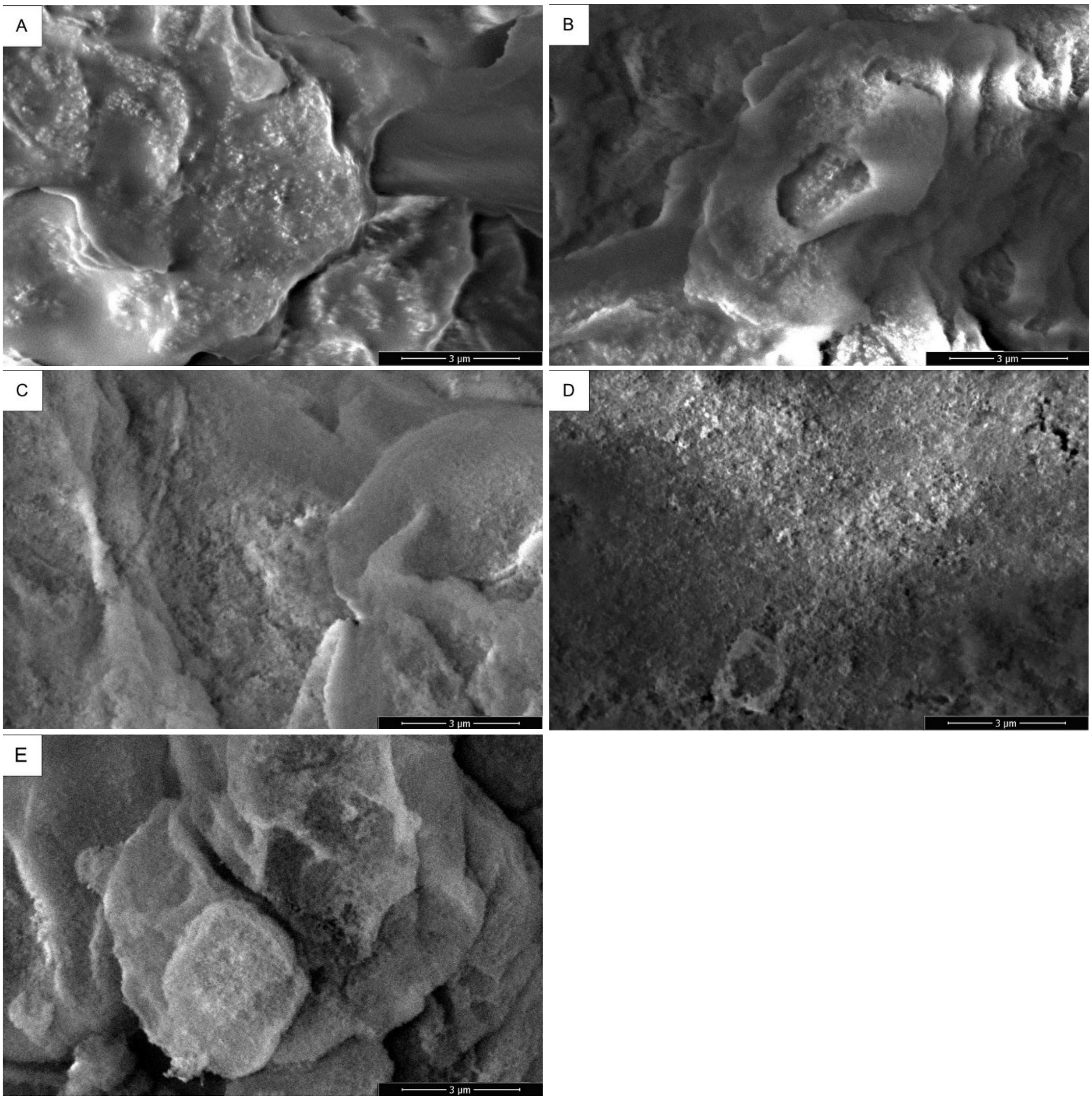

Figure S6 – SEM images of coatings based on a 10 wt.% poly(HMA-co-GMA) solution. showing variations in the filler/polymer binder ratios: A – 0.4; B – 0.8; C – 1.2; D – 1.6; E – 2.0 (30000× magnification)

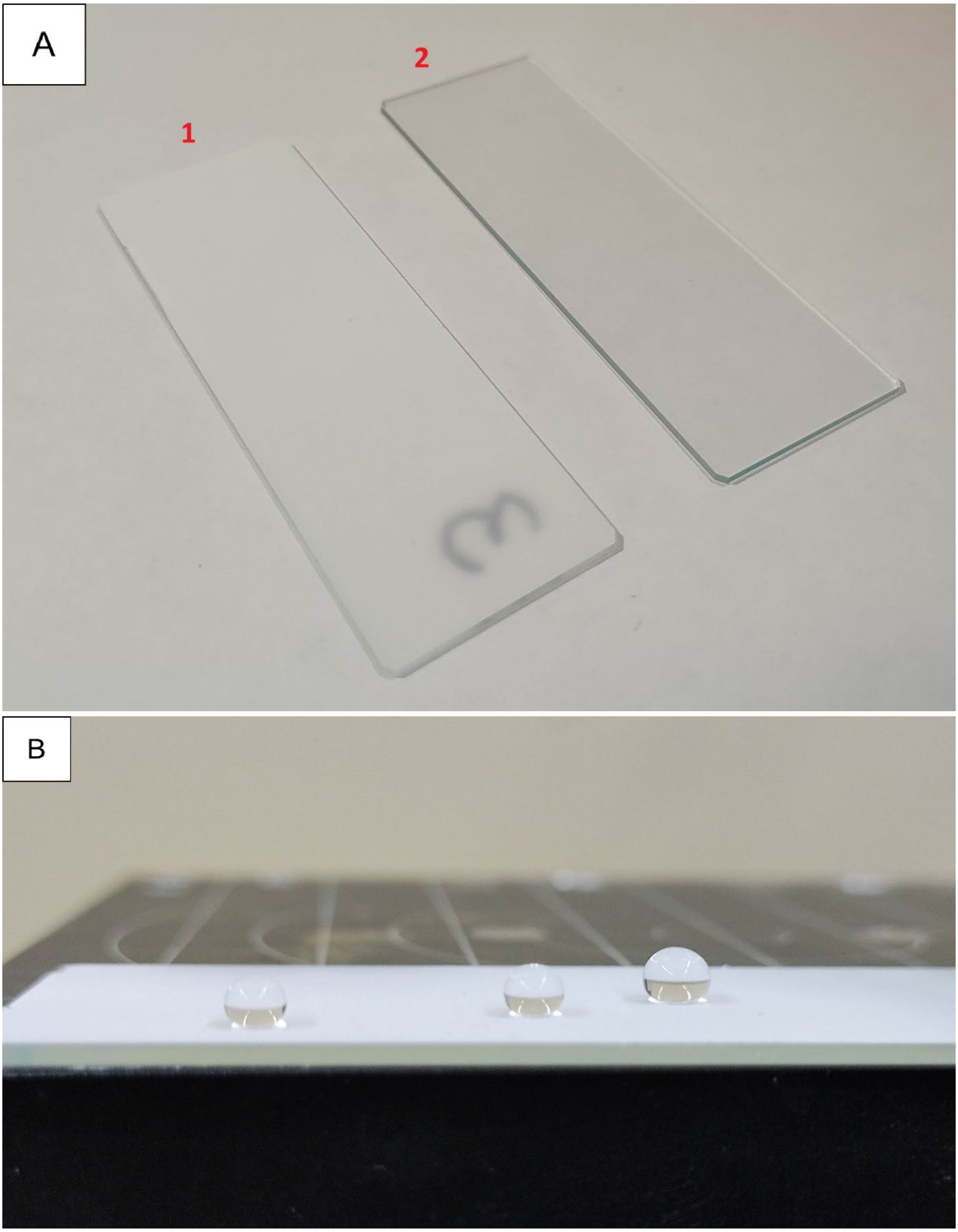
A
1
2
B

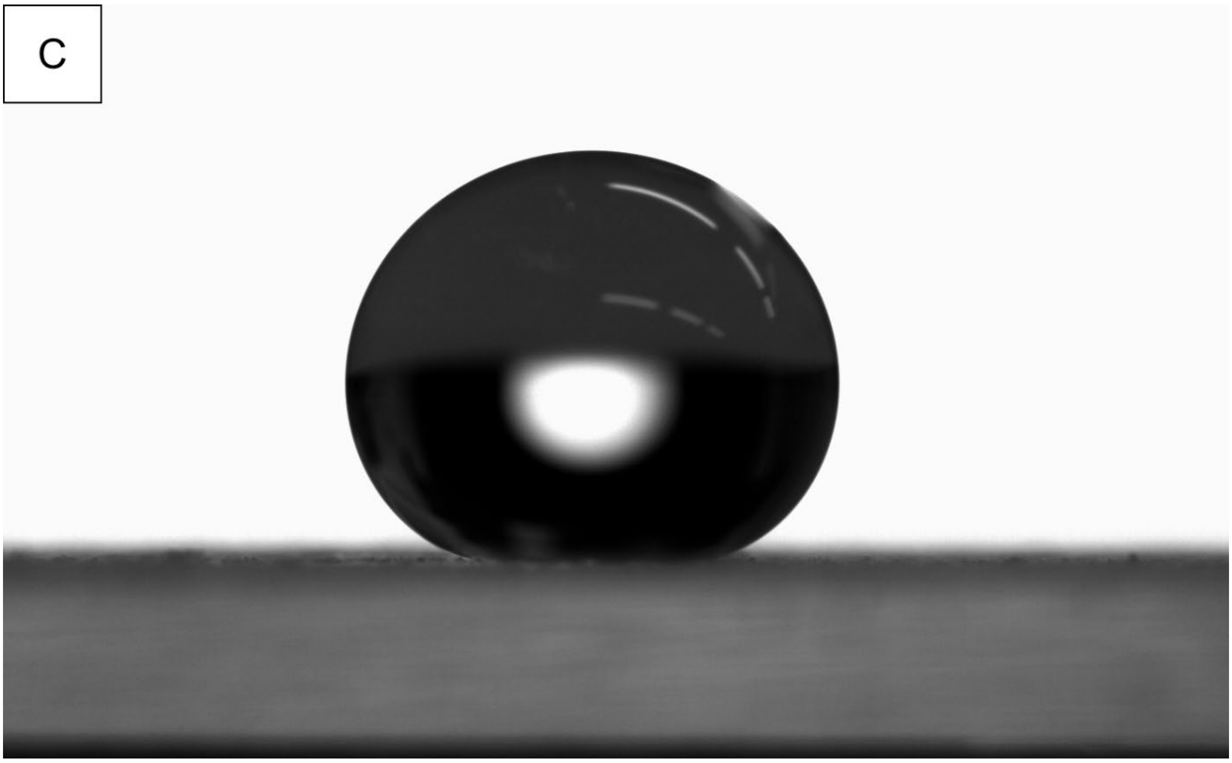

Figure S7 – Image of composite coating: A - uncoated glass (right) and coated glass (left) based on 5 wt.% poly(HMA-co-GMA) solution with filler/polymer binder ratio equal to 1.2 ; B - water droplets on the surface of composite coating; C - profile of water droplet on the surface of composite coating.
